# Phylogenetic analysis of the mitochondrial genomes in bees (Hymenoptera: Apoidea: Anthophila)

**DOI:** 10.1371/journal.pone.0202187

**Published:** 2018-08-09

**Authors:** Bo He, Tianjuan Su, Yupeng Wu, Jinshan Xu, Dunyuan Huang

**Affiliations:** 1 Chongqing Key Laboratory of Vector Insects, Chongqing Key Laboratory of Animal Biology, Chongqing Normal University, Chongqing, China; 2 Key Laboratory of Cultivation and Protection for Non-Wood Forest Trees, Ministry of Education, Central South University of Forestry and Technology, Changsha, Hunan, China; 3 Key Laboratory of Zoological Systematics and Evolution, Institute of Zoology, Chinese Academy of Sciences, Beijing, China; 4 College of Environment and Safety, Taiyuan University of Science and Technology, Taiyuan, Shanxi, China; Sichuan University, CHINA

## Abstract

In this study, the first complete mitogenome of Andrenidae, namely *Andrena camellia*, is newly sequenced. It includes 13 protein-coding (PCG) genes, 22 transfer RNA (rRNA) genes, two ribosomal RNA (tRNA) genes, and a control region. Among PCGs, high conservation is observed in cytochrome oxidase genes with *cox1* exhibits the highest conservation. Conversely, NADH dehydrogenase and ATPase subunit genes are more variable with *atp8* presents the maximal variation. Comparison of the gene order indicates complex rearrangement in bees. Most of the rearranged events are located in the tRNA clusters of *trnI*-*trnQ*-*trnM*, *trnW*-*trnC*-*trnY*, and *trnA-trnR-trnN-trnS1-trnE-trnF*. Furthermore, we present the most comprehensive mitochondrial phylogeny of bee families. The monophyly of each family and the long-tongued bees is highly supported. However, short-tongued bees are inferred as paraphyletic relative to the sister relationship between Melittidae and other bee families. Furthermore, to improve the resolution of phylogeny, various datasets and analytical approaches are performed. It is indicated that datasets including third codons of PCGs facilitate to produce identical topology and higher nodal support. The tRNA genes that have typical cloverleaf secondary structures also exhibit similar positive effects. However, rRNAs present poor sequence alignment and distinct substitution saturation, which result in negative effects on both tree topology and nodal support. In addition, Gblocks treatment can increase the congruence of topologies, but has opposite effects on nodal support between the two inference methods of maximum likelihood and Bayesian inference.

## Introduction

Bees (Hymenoptera: Apoidea: Anthophila) are widely distributed and comprise approximately 20,000 described species [1]. They are considered as the primary pollinators of angiosperm, and play an important role in natural and agricultural ecosystems [2–4]. Therefore, it is significant to have an accurate understanding of their phylogenetic relationships. However, the higher-level relationships of bees remain contentious, such as the basal lineage of bees and the relationships within short-tongued bees [1, 5–7].

**Fig 1:**
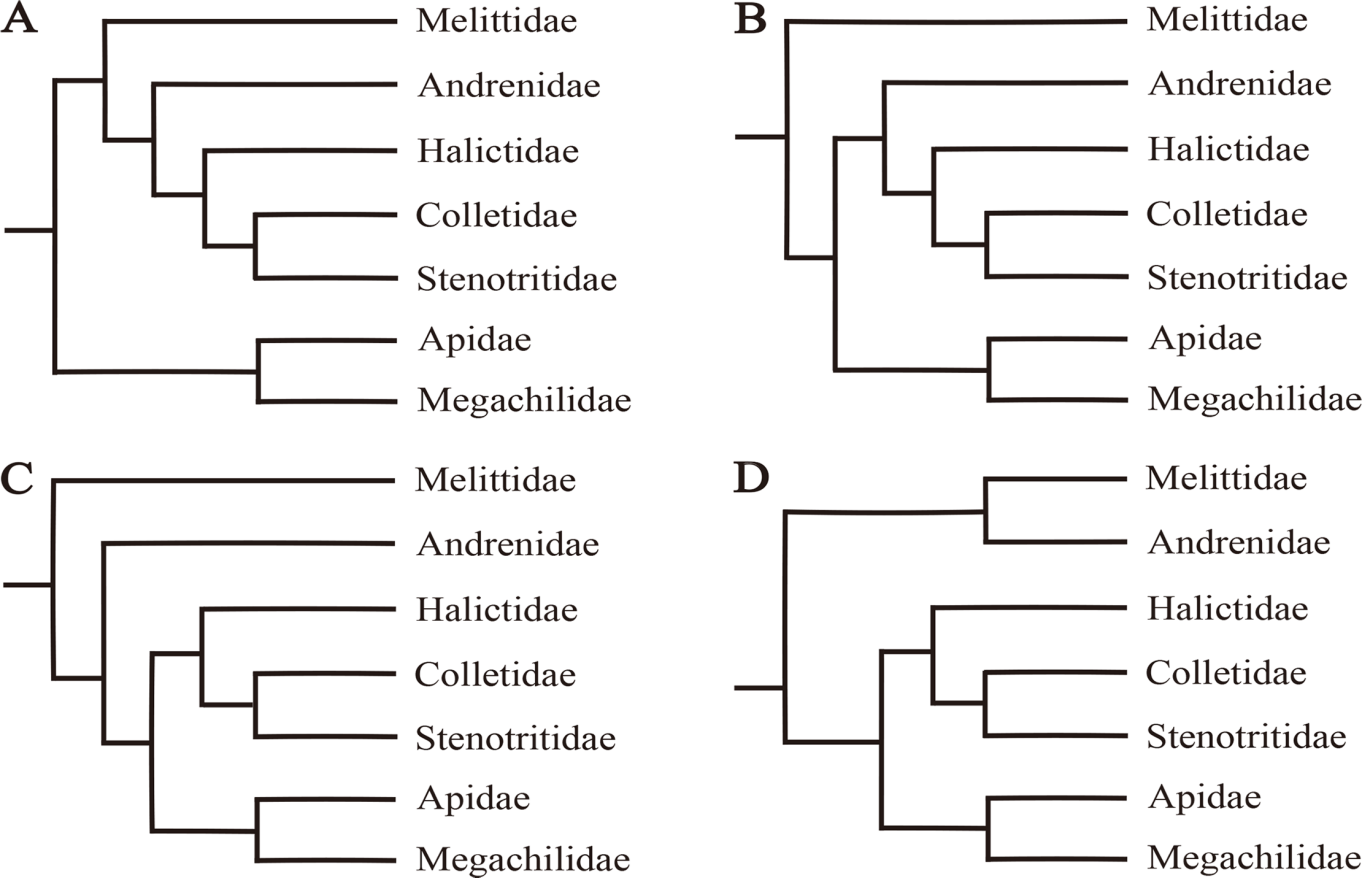
Previous phylogenetic analyses of bees. (A) bees were divided into long-tongued bees and short-tongued bees; (B) Melittidae was inferred as the basal lineage of bees or sister to other bee families; (C) and (D) Andrenidae was suggested as sister to all other bees except Melittidae or sister to Melittidae, respectively.

The extant bees are generally classified into seven families (Apidae, Megachilidae, Colletidae, Melittidae, Andrenidae, Halictidae, and Stenotritidae) [3]. The families Apidae and Megachilidae clearly form a monophyletic group (i.e. long-tongued bees) (Fig 1A) [3, 8] based on the shared morphological feature of highly modified first and second labial palpal segments. The remaining bee families are short-tongued bees, in which Colletidae had been proposed as the sister group to the remaining bee families [9]. However, some other studies suggested that Melittidae was either the sister to other bees, or a paraphyletic group from which all the remaining bees were derived (Fig 1B) [10–12]. In addition, Andrenidae had also been suggested as the sister to the group containing Halictidae, Colletidae, and Stenotritidae (Fig 1B), sister to all other bees except Melittidae (Fig 1C), or sister to Melittidae (Fig 1D) [13].

Although most studies based on morphology and nuclear genes suggested that Melittidae was sister to all other bee families, and the remaining bees were classified into two groups: (Apidae + Megachilidae) and (Andrenidae + (Halictidae + (Stenotritidae + Colletidae))) [3, 7, 12, 14–16], one recent study based on complete mitochondrial genomes (mitogenomes) presented the phylogenetic relationships of (Apidae + (Colletidae + Melittidae)) (only three bee families were analyzed) [17]. Owing to some unique features like the maternal inheritance, high copy numbers, strict orthologous genes, accelerated rate of nucleotide substitution, and low rate of recombination, mitogenome had been extensively used to infer phylogeny of insects [18–21]. Therefore, the conclusion proposed by Kahnt et al. [17] might be reasonable. The debate might be due, in part, to the unbalanced distribution of mitogenomes among bee families or the different evolutionary history between nuclear and mitochondrial genomes.

To date, only 12 complete mitogenomes have been sequenced for bees, (GenBank, December 1, 2017), and the corresponding phylogenetic analyses are still limited. In this study, we present the first complete mitogenome of Andrenidae (*Andrena camellia*). With all the complete and nearly complete mitogenomes of bees (62 species in total), a series of phylogenetic analyses were conducted to 1) estimate the suitability of mitogenomes for resolving higher-level relationships of bees and 2) assess the effects of different datasets, the Gblocks treatment, and the inference methods on mitochondrial phylogenetic analyses.

## Materials and methods

### Sample collection, PCR amplification, and sequences annotation

The specimen of *A*. *camellia* was collected in Xinyu City, Jiangxi Province, China. This species was identified by a taxonomic expert (Dr. Ze-qing Niu, Institute of Zoology, Chinese Academy of Sciences) using the traditional morphological approaches, with the voucher specimen (No. CAS-2015-4Y) preserved in absolute ethyl alcohol and stored at −20°C freezer in Institute of Zoology, Chinese Academy of Sciences until use. Total genomic DNA was extracted from legs of the single sample using the DNeasy Blood & Tissue kit (Qiagen Hilden, Germany) following the manufacturer’s instructions.

Ten pairs of primers were used (S1 Table), some of which were universal [22, 23]. Specific primers were designed according to the initially sequenced fragments. PCR was performed under the following conditions: 2 min at 92°C, followed by 40 cycles of 30 s at 92°C, 30 s at 48–55°C, and 12 min at 60°C, and a final extension at 60°C for 20 min. The PCR products were detected by electrophoresis in 1% agarose gel, purified using the 3Spin PCR Product Purification Kit, and sequenced using BigDye v3.1 with a DNA sequencer of ABI 3730XL (PE Applied Biosystems, San Francisco, CA, USA). Additionally, in order to generate high-quality sequences, some of the purified PCR products were also ligated to the pUCm-T vector (Sangon Biotech, China). Multiple clones were independently sequenced.

The overlapping PCR products were assembled using SeqMan program included in the Lasergene software package (DNAStar Inc., Madison, Wisc.). The transfer RNA genes (tRNAs) were predicted using the Mitos WebServer [24], with the Mito genetic code of invertebrate. The positions of protein-coding genes (PCGs), ribosomal RNA genes (rRNAs), and the control region were confirmed by the boundaries of tRNAs and by comparing with sequences from closely related species. To ensure the accuracy of the nucleotide sequences of PCGs, each of which was also translated into amino acids according to the invertebrate mitochondrial genetic code.

### Comparative analysis of the mitogenomes

A total of 64 species were analyzed in this study, including 62 bees and two outgroups from the families Vespidae (*Abispa ephippium*) and Crabronidae (*Philanthus triangulum*) (S2 Table). The comparable gene identity map was visualized by the CGView Comparison Tool [25]. The mitogenomes that composed of two or more sequence fragments were excluded from the analysis. Features of gene arrangement were also performed on mitogenomes that having the whole typical set of 37 genes and the control regions only. In addition, species belonged to the same genus or subspecies from the same species, sharing the identical gene rearrangement events, were represented by only one mitogenome. The base composition was calculated by MEGA 6.05 [26]. Composition skew analysis was calculated according to the formulas: AT-skew = (A-T)/(A+T) and GC-skew = (G-C)/(G+C) [27]. Potential saturation of PCGs, rRNAs, and tRNAs was assessed using the index of substitution saturation (*Iss*) implemented in DAMBE 6.1.17 [28].

### Sequence alignment

Nucleotide sequences for each of the 13 PCGs, two rRNAs, and 22 tRNAs were imported into separate files using BioEdit 7.1.3.0 [29]. For PCGs (excluding the stop codons), the amino acid alignment was generated for each gene and aligned separately using Muscle implemented within MEGA. The corresponding nucleotide alignments were then toggled back from the amino acid alignments. The rRNAs and tRNAs were aligned with MAFFT 7.310 using the Q-INS-i algorithm [30].

To eliminate poorly aligned positions and divergent regions, Gblocks 0.91b [31] was used with the following settings: For PCGs, default parameters were set except for the gap positions toggled as “all”, which meant that all gap positions could be selected; For rRNAs and tRNAs, which had many small but conserved blocks, the relaxed parameter settings were performed (“gap positions” allowed as “all”; other parameters including “minimum number of sequence for a conserved position”, “minimum number of sequence for a flank position”, and the “minimum length of a block” were set as “minimum”).

### Datasets and substitution model selection

In order to test the effects of the third codon positions, gene types, and the Gblocks treatment on phylogeny, 16 datasets were carried out: 1) all codon positions of PCGs, with the Gblocks treatment (P123_G); 2) P123 and rRNAs (P123R_G); 3) P123 and tRNAs (P123T_G); 4) P123, rRNAs, and tRNAs (P123RT_G); 5) first and second codon positions of PCGs (P12_G); 6) P12R_G; 7) P12T_G; 8) P12RT_G; 9) P123 analyzed without Gblocks (P123_UnG); 10) P123R_UnG; 11) P123T_UnG; 12) P123RT_UnG; 13) P12_UnG; 14) P12R_UnG; 15) P12T_UnG; 16) P12RT_UnG. The best partitioning schemes and nucleotide substitution models were simultaneously confirmed with PartitionFinder 2.1.1 [32] using the Bayesian Information Criterion (BIC). The data blocks for each dataset were pre-defined by both gene types (each of 13 PCGs, two rRNAs, and 22 tRNAs) and codon positions (first, second, and third codon positions for each PCG).

### Phylogenetic inference

Two inference methods, maximum likelihood (ML) and Bayesian inference (BI), were performed using RAxML 8.2.9 [33] and MrBayes 3.2.6 [34], respectively, through the online CIPRES Science gateway [35]. For the ML analyses, *A*. *ephippium* and *P*. *triangulum* were selected as outgroups, and 1,000 bootstrap replicates were conducted with the GTRGAMMA model applied to all partitions. In the BI, *A*. *ephippium* was selected as the outgroup. Two independent runs were performed, each with three hot chains and one cold chain. Posterior distributions were estimated using Markov Chain Monte Carlo (MCMC) sampling. The MCMC chains were set for 10,000,000 generations, with sampling every 1,000 steps and a burn-in process for the first 25% steps.

## Results

### Genome structure and organization

**Fig 2:**
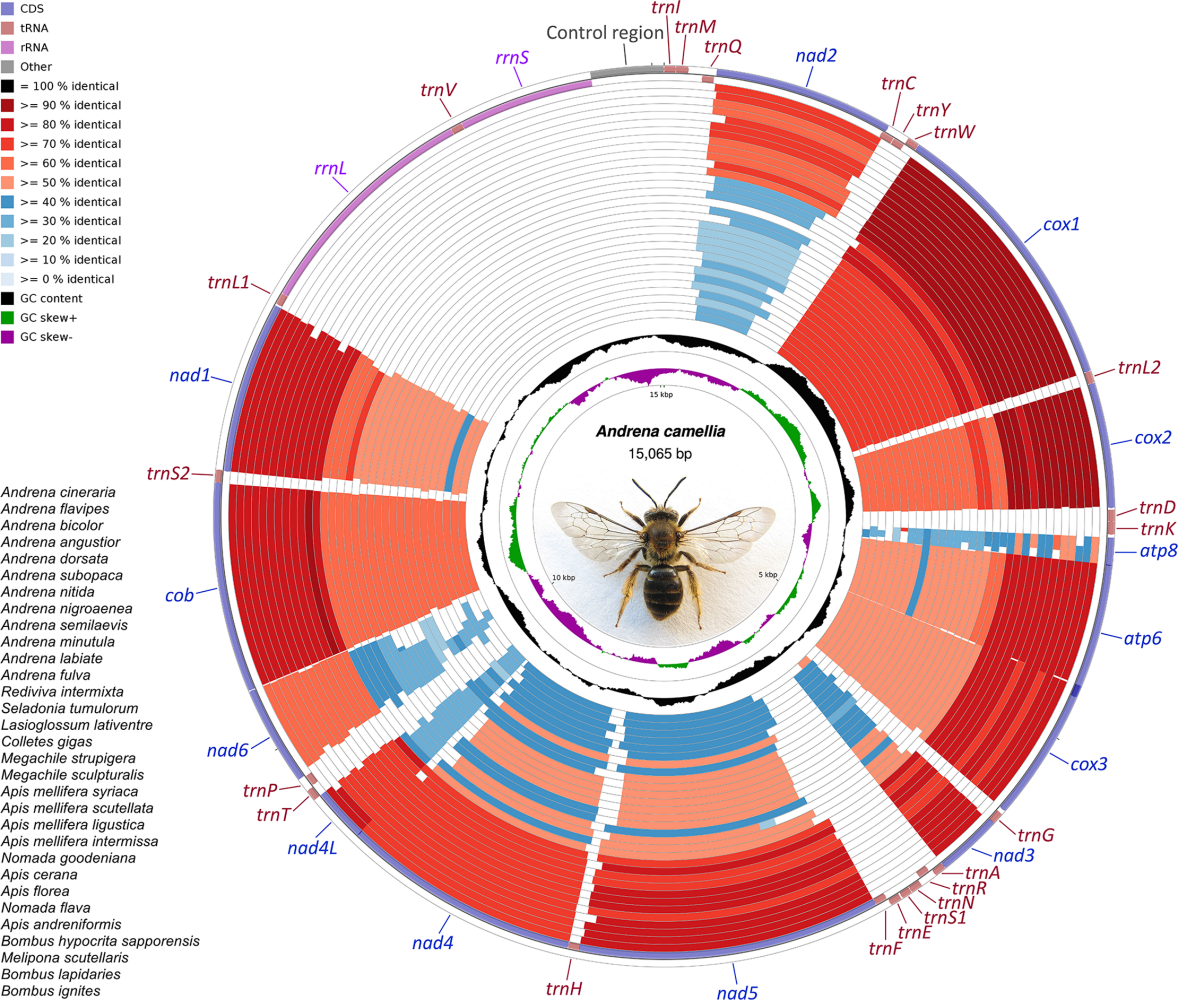
Circular map of the mitogenomes of bees. Gene identity is obtained by BLAST searches, with the reference genome of *A*. *camellia*. The sequences are arranged in an order that the most similar mitogenome is closest to the outer edge of the map.

The mitogenome of *A*. *camellia* (GenBank accession KX241615) was completely sequenced, with the length of 15,065 bp. It contained the typical set of 37 genes, including 13 protein-coding genes (PCGs), two rRNAs, and 22 tRNAs. Except for the control region, 16 intergenic spaces (181 bp in total) and 12 overlapping regions (63 bp in total) were dispersed throughout the whole genome. Twenty-two genes were coded on the J-strand, the other fifteen genes were coded on the N-strand (Fig 2). The nucleotide composition of *A*. *camellia* was biased toward A + T (78.58%) (S3 Table). However, it showed the lowest A+T content within all complete mitogenomes of bees analyzed. In addition, nearly complete mitogenomes of other *Andrena* species also presented the relatively low A + T content, such as *A*. *cineraria* (78.82%), *A*. *semilaevis* (76.63%), and *A*. *dorsata* (75.31%) (S3 Table). The nucleotide skewness (AT-skew = 0.170, GC-skew = -0.373) indicated strong A-skew and C-skew. Comparative analyses of other bees also showed that most of the AT-skews were positive, while most GC-skews were negative (S3 Table).

To better visualize the gene identity in mitogenomes of bees, the comparable circular map was generated (Fig 2). Given the rearrangement of tRNAs, the gene identity map was drawn based on the PCGs alone. Pairwise comparisons of the concatenated PCGs between *A*. *camellia* and other bee species revealed the highest similarity between *A*. *camellia* and other species of Andrenidae, followed by Melittidae, Halictidae, and Colletidae. Conversely, relative low similarity was observed between *A*. *camellia* and long-tongued bees (i.e. Megachilidae and Apidae). High conservation was observed in cytochrome oxidase genes with *cox1* exhibited the highest conservation. Conversely, NADH dehydrogenase and ATPase subunit genes were more variable with *atp8* presented the maximal variation.

### Gene rearrangement

Gene rearrangement could be classified into local inversion (inverted in the local position), remote inversion (translocated and inverted), gene shuffling (local translocation), and translocation [36]. Local inversion had been proposed as a major type of gene rearrangement in mitogenomes of Hymenoptera [37]. However, it was found that gene shuffling (*trnQ*/*trnM*, *trnW*/*trnC*-*trnY*, and *trnK*/*trnD*) was dominant in the mitogenome of *A*. *camellia*. In addition, a local inversion of *trnR* was also detected.

**Fig 3:**
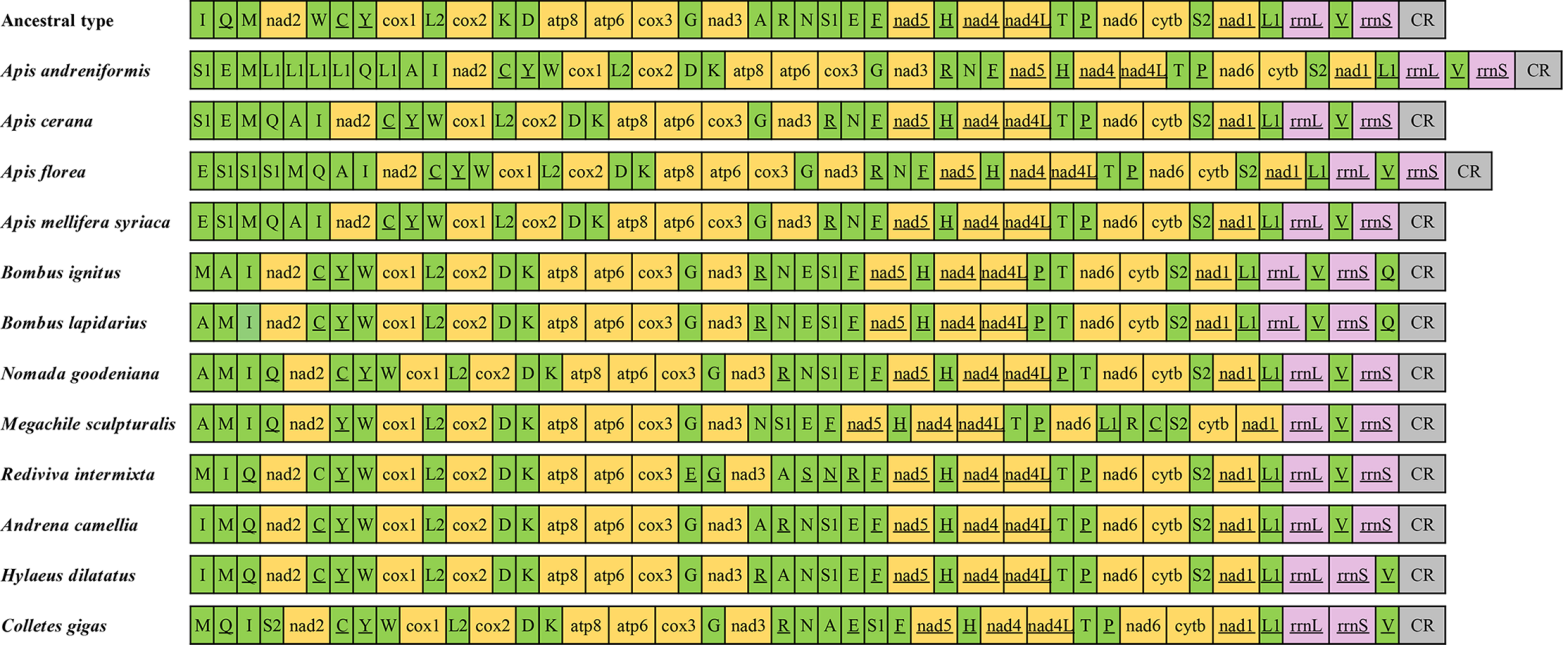
Gene arrangement of the mitogenomes of bees. PCGs, rRNAs, tRNAs, and the control region are marked with yellow, pink, green, and grey, respectively. Gene with underscore indicates that it is encoded in the N strand.

Compared with the putative ancestral gene arrangement of insects (Fig 3), all the complete or nearly complete mitogenomes of bees presented gene rearrangements, with species from the same genus (e.g. *Andrena*) or subspecies from the same species (e.g. *Apis mellifera*) sharing the identical gene rearrangement events. There was no PCG or rRNA rearrangement in the bee mitogenomes. The rearranged tRNAs were mainly located in the tRNA clusters of *trnI*-*trnQ*-*trnM*, *trnW*-*trnC*-*trnY*, and *trnA-trnR-trnN-trnS1-trnE-trnF*, which were also reported as the rearrangement hot spots in the mitogenomes of Hymenoptera [36, 38].

### Substitution saturation tests

Substitution saturation index (*Iss*) for the first and second codons in PCGs was significantly smaller than the critical value (*Iss*.*cSym* and *Iss*.*cAsym*) (Table 1). The *Iss* value of the third codons of PCGs and all sites of tRNAs was larger than the *Iss*.*cAsym*, but smaller than the *Iss*.*cSym*, which indicated that the third codons of PCGs and tRNAs might provide poor information for phylogenetics under the assumption of a very asymmetrical true tree, but would provide useful information for a symmetrical true tree. Notably, the *Iss* value of rRNAs was significantly larger than both the *Iss*.*cSym* and the *Iss*.*cAsym*. It was indicated that rRNAs had experienced substitution saturation and might provide poor information for phylogenetics under the assumption of both a very symmetrical and a very asymmetrical true tree.

**Table 1:** Saturation substitution tests for PCGs, rRNAs, and tRNAs of mitogenomes of bees.

	Gene regions	NumOTU	*Iss*	*Iss*.*cSym*	Psym	*Iss*.*cAsym*	Pasym
**UnGblocks**	**1st codons**	32	0.517	0.809	0.0000	0.554	0.0001
	**2nd codons**	32	0.518	0.809	0.0000	0.554	0.0008
	**3rd codons**	32	0.751	0.809	0.0000	0.554	0.0000
	**All codons**	32	0.533	0.818	0.0000	0.572	0.0000
	**rRNAs**	32	0.874	0.802	0.0000	0.539	0.0000
	**tRNAs**	32	0.704	0.79	0.0000	0.52	0.0000
**Gblocks**	**1st codons**	32	0.484	0.808	0.0000	0.551	0.0000
	**2nd codons**	32	0.348	0.808	0.0000	0.551	0.0000
	**3rd codons**	32	0.735	0.808	0.0000	0.551	0.0000
	**All codons**	32	0.496	0.817	0.0000	0.571	0.0000
	**rRNAs**	32	0.774	0.777	0.7069	0.496	0.0000
	**tRNAs**	32	0.634	0.782	0.0000	0.507	0.0000

Notes: NumOTU, number of OTUs; *Iss*, the index of substitution saturation; *Iss*.*cSym*, critical *Iss* based on a perfectly symmetrical tree topology; *Iss*.*cASym*, critical *Iss* based on an extremely asymmetrical tree topology [39]; Psym/Pasym, probability

### Methodological effects of various approaches

In order to test the effects of gene types, the combined analyses of PCGs + rRNAs, PCGs + tRNAs, and PCGs + rRNAs + tRNAs were compared with PCGs alone (Table 2). Inclusion of rRNAs had negative effects on nodal support at least when the third codons of PCGs included (e.g. P123R_G_BI vs. P123_G_BI), and had negative effects on both topology and nodal support at most when the datasets excluding third codons were analyzed (e.g. P12R_UnG_ML vs. P12_UnG_ML). However, tRNAs always had positive effects on nodal support (e.g. P123RT_G_BI vs. P123R_G_BI). Notably, all datasets including third codons of PCGs produced the identical topology and relatively high nodal support (e.g. P123_UnG_BI vs. P12_UnG_BI).

**Table 2:** Summary of the major clades recovered by different datasets and analytical approaches.

Clade	P123		P12		P123R		P12R		P123T		P12T		P123RT		P12RT	
	G	UnG	G	UnG	G	UnG	G	UnG	G	UnG	G	UnG	G	UnG	G	UnG
**RAxML (ML)**																
**Mel+other bees**	100	100	100	100	100	100	100	100	100	100	100	100	100	100	100	100
**LT-bees+ST-bees(ME)**	98	99	98	98	96	98	97	98	99	98	99	97	98	100	99	98
**Apidae+Megachilidae**	100	100	100	100	100	100	100	100	100	100	100	100	100	100	100	100
**Halictidae+(And+Col)**	-	-	-	-	-	-	-	-	-	-	-	-	-	-	-	-
**Andrenidae+(Hal+Col)**	99	99	94	96	99	100	99	-	100	100	98	98	100	100	100	-
**Colletidae+(Hal+And)**	-	-	-	-	-	-	-	99	-	-	-	-	-	-	-	100
**Andrenidae+Colletidae**	-	-	-	-	-	-	-	-	-	-	-	-	-	-	-	-
**Halictidae+Colletidae**	57	47	42	39	50	40	40	-	60	54	51	51	56	40	43	-
**Halictidae+Andrenidae**	-	-	-	-	-	-	-	36	-	-	-	-	-	-	-	42
**Halictidae**	100	100	100	100	100	100	100	100	100	100	100	100	100	100	100	100
**Andrenidae**	100	100	100	100	100	100	100	100	100	100	100	100	100	100	100	100
**Colletidae**	84	81	86	83	97	98	99	99	91	88	94	94	99	99	100	99
**Apidae**	100	100	100	100	100	100	100	100	100	100	100	100	100	100	100	100
**Megachilidae**	100	100	100	100	100	100	100	100	100	100	100	100	100	100	100	100
**MrBayes (BI)**																
**Mel+other bees**	1	1	1	1	1	1	1	1	1	1	1	1	1	1	1	1
**LT-bees+ST-bees(ME)**	1	1	1	1	1	1	1	1	1	1	1	1	1	1	1	1
**Apidae+Megachilidae**	1	1	1	1	1	1	1	1	1	1	1	1	1	1	1	1
**Halictidae+(And+Col)**	-	-	-	-	-	-	1	1	-	-	-	-	-	-	-	-
**Andrenidae+(Hal+Col)**	1	1	1	1	1	1	-	-	1	1	1	1	1	1	1	-
**Colletidae+(Hal+And)**	-	-	-	-	-	-	-	-	-	-	-	-	-	-	-	-
**Andrenidae+Colletidae**	-	-	-	-	-	-	0.66	0.73	-	-	-	-	-	-	-	-
**Halictidae+Colletidae**	0.98	0.99	0.68	0.53	0.74	0.88	-	-	0.99	1	0.86	0.93	0.93	0.98	0.69	-
**Halictidae+Andrenidae**	-	-	-	-	-	-	-	-	-	-	-	-	-	-	-	-
**Halictidae**	1	1	1	1	1	1	1	1	1	1	1	1	1	1	1	1
**Andrenidae**	1	1	1	1	1	1	1	1	1	1	1	1	1	1	1	1
**Colletidae**	1	1	1	1	1	1	1	1	1	1	1	1	1	1	1	1
**Apidae**	1	1	1	1	1	1	1	1	1	1	1	1	1	1	1	1
**Megachilidae**	1	1	1	1	1	1	1	1	1	1	1	1	1	1	1	1

Notes: G, usage of Gblocks; UnG, without Gblocks; -, not recovered; LT-bees, long-tongued bees; ST-bees, short-tongued bees; ME, Melittidae excluded; Mel, Melittidae; Hal, Halictidae; And, Andrenidae; Col, Colletidae; P123, all codon positions of PCGs; P12, first and second codon positions of PCGs; P123R, P123 and rRNAs; P12R, P12 and rRNAs; P123T, P123 and tRNAs; P12T, P12 and tRNAs; P123RT, indicates P123, rRNAs, and tRNA; P12RT, indicates P12, rRNAs, and tRNAs.

Gblocks generally reduced the degree of substitution saturation and presented positive effects on tree topology, but had opposite effects on nodal support between the two inference methods. Under the ML framework, datasets with Gblocks treatment presented the same tree topology and had much higher nodal support. In BI, P12RT that analyzed without Gblocks treatment even failed to present a clear relationship among Andrenidae, Halictidae, and Colletidae. However, the nodal support of Halictidae + Colletidae generated with Gblocks was slightly lower.

The two inference methods produced the same tree topology, except for the analyses of P12R_G, P12R_UnG, and P12RT_UnG. It was indicated that when rRNAs excluded, the tree topology showed little sensitivity to inference methods. In addition, compared with ML method, the BI generally presented higher nodal support, such as the node of Halictidae + Colletidae.

### Phylogeny

**Fig 4:**
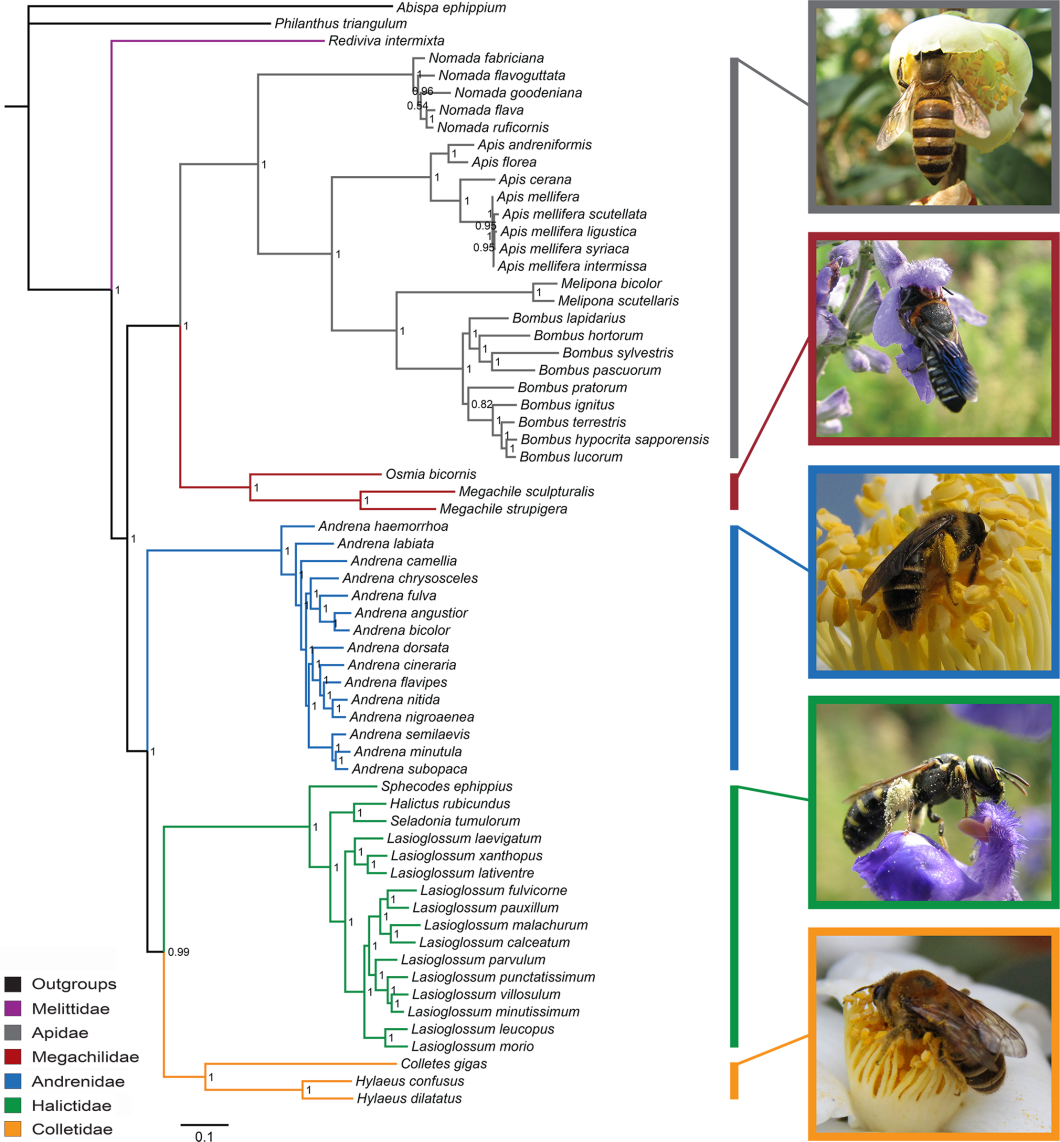
The phylogenetic relationships of bees inferred from the mitogenome dataset of P123T_G_BI. Numbers on branches are Bayesian posterior probabilities.

Phylogenetic analyses were performed on 64 complete or nearly complete mitogenomes, representing six bee families. In order to assess the effects of datasets (based on codons and gene types), Gblocks treatment (Gblocks and UnGblocks), and inference methods (BI and ML), a total of 32 independent phylogenetic analyses were carried out. Based on the relationships among bee families, four different tree topologies were recovered. As shown in Table 2, some nodes were consistently recovered by all analyses. For example, the monophyly of each family was robustly supported except for Melittidae (only one species could be retrieved from GenBank). Given that the sister relationship between Melittidae and other bee families was highly supported in all datasets, the short-tongued bees were inferred as a paraphyletic group (Fig 4). However, the monophyly of long-tongued bees was highly supported. The argument was only presented in short-tongued bee families. Among the 32 independent data analyses, 27 of which presented the relationship of (Andrenidae + (Halictidae + Colletidae)). However, the relationship of Halictidae + (Andrenidae + Colletidae) was generated from the analyses of P12R_G_BI and P12R_UnG_BI. The Colletidae + (Halictidae + Andrenidae) was produced by the analyses of P12R_UnG_ML and P12RT_UnG_ML. The unresolved relationship of Andrenidae + Halictidae + Colletidae was also inferred from the analysis of P12RT_UnG_BI.

## Discussion

### Methodological effects of various approaches

The effect of RNA genes on tree topology and nodal support is a long-standing debate [18]. The rRNAs and tRNAs, which comprise about 15% and 10% of the genic sequence, respectively, were often excluded in phylogenetic reconstruction of insects [20]. However, other studies suggested that rRNAs and tRNAs could improve nodal confidence and the stabilization of highly variable backbone relationships [18, 40, 41]. In our study, inclusion of rRNAs led to more variable and poorly supported phylogenetic relationships. However, this effect could be eliminated by the inclusion of the third codons of PCGs and be reduced by tRNAs. One possible reason for the noisy signals in rRNAs might be the challenge of accurate alignments. Although combining secondary structural information with alignments was supposed to increase the accuracy, it was difficult and time-consuming to apply for more remotely related taxa [42]. Another candidate explanation might be the substitution saturation (Table 1), indicating poor phylogenetic signals existed in rRNAs. By contrast, inclusion of tRNAs resulted in more consistent topologies and relatively high support value. Although tRNAs were very short (58–72 bp for each tRNA in *A*. *camellia*), they had typical cloverleaf secondary structures (S1 Fig). Such conservative structures would facilitate more accurate alignments. In addition, the *Iss* value of tRNAs was smaller than the *Iss*.*cSym*, though larger than *Iss*.*cAsym*, indicating that tRNAs might provide useful information for a more symmetrical tree.

Whether to include the third codons is also an ongoing debate. Some studies proposed that exclusion of third codons could produce more consistent topologies [20, 43]. Other studies suggested that the third codons which contributed valuable phylogenetic signals for reconstructing phylogenetic relationships should be included [44–46]. In this study, inclusion of third codons positively increased the nodal support and produced the identical tree topology (Table 2). Therefore, the third codons of PCGs should be assessed objectively and should not be eliminated directly. Given the different effects of third codons on phylogeny, it would be standard practice to evaluate within each taxonomic scale the effects of including or excluding of third codons on topology and nodal support [18]. Furthermore, the degree of substitution saturation should also be considered as a useful measurement. In our present analyses, although the *Iss* value of the third codons was larger than the *Iss*.*cAsym*, it was smaller than the *Iss*.*cSym*, which indicated that the third codons might provide valuable information under the hypothesis of a more symmetrical tree.

Based on algorithm, Gblocks was used to increase signal-to-noise ratio by eliminating poorly aligned positions and highly divergent regions. These regions might be nonhomologous or include inaccurately defined gene boundaries, or have been saturated by multiple substitutions [31, 47]. For example, alignment of *nad5* genes in bee species showed that *Lasioglossum punctatissimum* was shorter than all other species at the 5*'* end (e.g. 84 bp shorter than *Hylaeus dilatatus*). Similar problems had also been found in genes of *cox1*, *nad2*, *nad4*, *nad4l*, and *nad5* of some owlet moths [41]. Therefore, the gene boundaries, especially for 5*'* end, might include some relatively arbitrary definition. However, a comparative analysis suggested that eliminating variable regions presented a negative effect on phylogenetic accuracy [45]. Our studies showed that Gblocks had positive effects on both tree topology and nodal support in ML analyses. By contrast, with Gblocks treatment, although the tree topologies were positively affected, slightly poorer nodal support was generated in BI analyses. Similar conclusions were also drawn by other researchers [41, 48], and they suggested that different inference methods might be possessing different criteria for treating gaps, which would facilitate to form the slightly different effects on phylogeny.

For the same dataset, especially when the third codons of PCGs included, the tree topology had low sensitivity to inference methods. The nodal support in BI was generally higher than in ML. However, it had also been suggested that posterior probabilities were somewhat liberal [41, 49, 50]. Therefore, it was necessary to use different inference methods to assess the phylogenetic signals. Although the nodal support in ML presented lower, the reason might be that the software of RAxML allowed for only a single model (GTR, GTR+G, or GTR+I+G) of rate heterogeneity in partitioned analyses. However, there were always different kinds of best models for different partitions, such as the HKY model. Nevertheless, the consistent topology inferred from BI and ML somewhat confirmed the higher-level relationships among bee families.

### Phylogeny

In this study, we presented the most comprehensive mitochondrial phylogeny of the family-level relationships of bees. Our analyses highly supported the monophyly of each family, except for Melittidae which had only one species analyzed. This was consistent with other morphological and molecular studies [1]. We provided further evidence that Melittidae was sister to the remaining bee families, with the maximum likelihood bootstrap (BP) support of 100 and Bayesian posterior probability (PP) of 1.0. This relationship had been proposed in some multi-gene studies [7, 13, 14]. Analyses based on divergence time also got the identical conclusion [12]. However, one study using the paralogs of elongation factor 1-alpha (EF-1α) confirmed that the root of bees was only partially resolved, indicating a three-way split among Melittidae, Andrenidae, and the remaining bees [6]. Their result might be due to lack of resolution from insufficient DNA sequence. In addition, a recent study supported the sister relationship between Melittidae and Colletidae with the datasets of mitogenomes [17]. The possible reason might be the deficiency of mitogenome data (only three bee families available). Therefore, the sister relationship between Melittidae and other bee families, which was strongly supported by more taxa in this study, might be more reasonable. The other bee families were divided into two groups: (Apidae + Megachilidae) and (Andrenidae + (Halictidae + Colletidae)).

The monophyly of long-tongued bees was highly supported (BP = 100, PP = 1.0), which had also been supported by both morphological and other molecular studies [1]. Given sister relationship between Melittidae and other bee families, the short-tongued bees were inferred as a paraphyletic group. Although one study using three nuclear genes presented the monophyly of short-tongued bees [10], most morphological or molecular studies supported the paraphyly of short-tongued bees [9, 11, 12]. Furthermore, we robustly recovered the relationship of (Andrenidae + (Halictidae + Colletidae)) within short-tongued bees, which was generally consistent with recent studies performed on nuclear genes. For example, with 20 nuclear genes and over 1300 bees, Hedtke et al. [14] suggested that Andrenidae was sister to (Halictidae + Colletidae + Stenotritidae), although with weak bootstrap proportion. In addition, a phylogenomic analysis of ants, bees, and stinging wasps [15], and a study focused on the evolutionary history of Hymenoptera [16] also supported the relationship of (Andrenidae + (Halictidae + (Colletidae + Stenotritidae))).

This study firstly presented a comprehensive mitochondrial phylogeny of the family-level relationships of bees. As described above, the tree topology described in Fig 1B was supported by our analyses. Many studies proposed that incongruent phylogenetic signals were commonly found between nuclear and mitochondrial genes [51–53]. However, the mitochondrial phylogeny of bees in our analyses exhibited promising congruence with most of the molecular studies [11, 12, 15, 16], suggesting that mitogenomes were suitable for resolving higher-level relationships within bees. Furthermore, the comparative analyses of methodological effects of various approaches have also been provided. It was indicated that mitogenomes would have better performance if the alignments were detailed partitioned, with suitable evolutionary model for each data block. The careful evaluation of which data to include was also important. However, although this study presented the most comprehensive mitochondrial phylogeny of the bee families to date, mitogenomes of Stenotritidae was not available. Therefore, a denser taxon sampling is still needed for future studies.

## Supporting information

S1 Fig Putative secondary structures of the 22 tRNAs in the mitogenome of *A. camellia*. (PNG)

S1 Table Regions and primers in this study. (DOCX)

S2 Table List of 64 species in this study. (DOCX)

S3 Table Nucleotide composition of the mitogenomes of 64 species. (DOCX)
