# Supplementary figures and images for: Phylogenetic analysis of the mitochondrial genomes in bees (Hymenoptera: Apoidea: Anthophila)
Source: PLoS One. 2018 Aug 9;13(8):e0202187. doi: 10.1371/journal.pone.0202187 (PMC6084986; doi:10.1371/journal.pone.0202187)

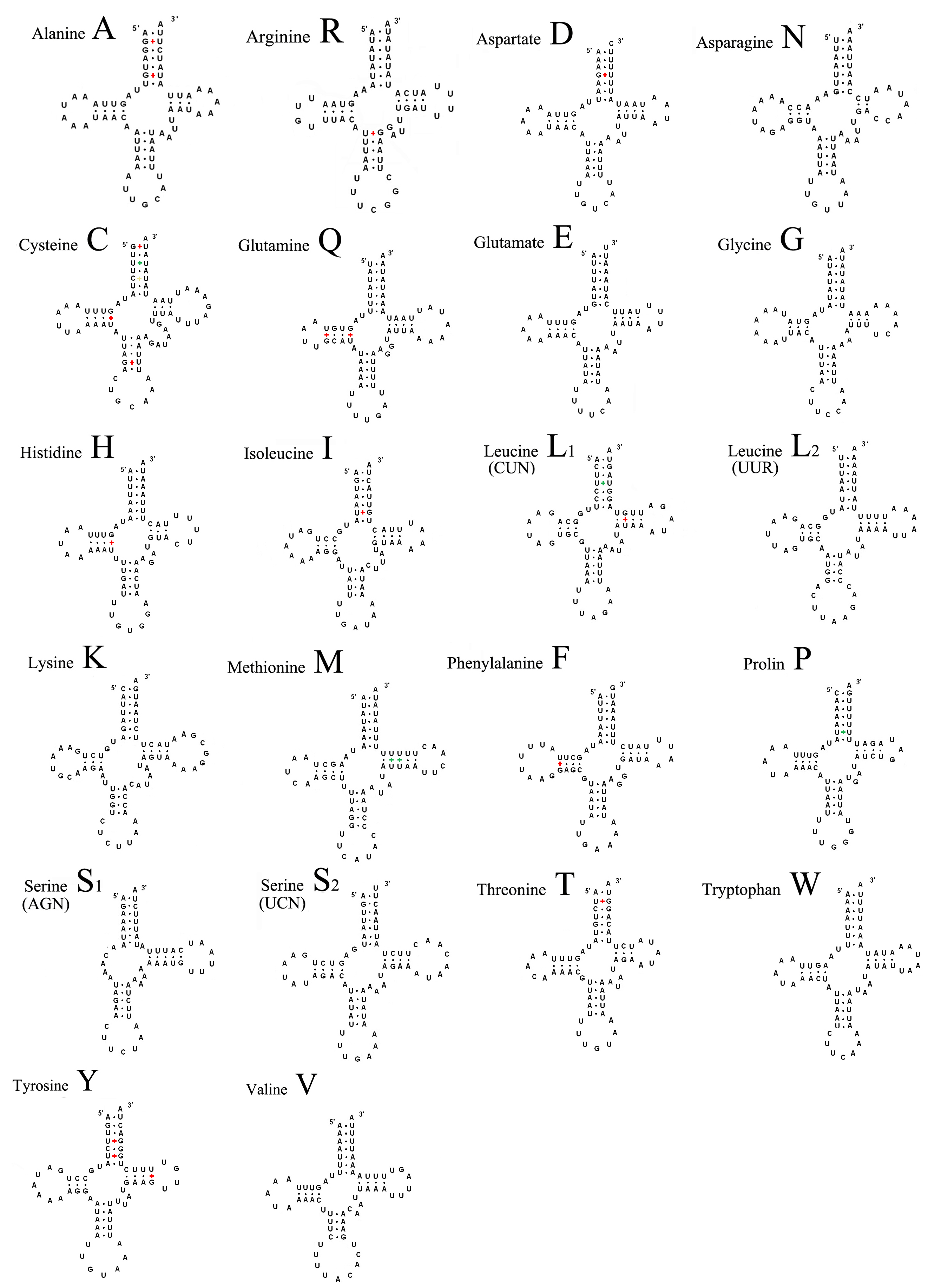

Supplement: S1 Fig — (PNG) [file pone.0202187.s001.png]
