# Supplementary material for: Phylogenetic analysis of the mitochondrial genomes in bees (Hymenoptera: Apoidea: Anthophila)
Source: PLoS One. 2018 Aug 9;13(8):e0202187. doi: 10.1371/journal.pone.0202187 (PMC6084986; doi:10.1371/journal.pone.0202187)
Supplement: S1 Table — (DOCX) [file pone.0202187.s002.docx]

**Table S1. Regions and primers in this study.**

| Region | Rrimer pair (F/R) | Primer sequence (F/R) 5'→3' |
| --- | --- | --- |
| *trnI-cox1* | TI-J34/4-C1-N-1.15d | GCCTGATAAAAAGGRTTAYYTTGATA/TGTTTCTTTTTTTCCTCTTTCAT |
| *cox1-trnL2* | C1-J-1751/TL2-N-3014 | GGATCACCTGATATAGCATTCCC/TTCAATGCACTTATTCTGCCATATTA |
| *cox1-atp8* | C1-J2756/A8-N4061 | ACATTCTTTCCTCARCAYTT/GAGAATAAGTTWGTTATCATTTTCA |
| *cox2-cox3* | C2-J-3696/C3-N-5460 | GAAATTTGTGGAGCAAATCATAG/TCAACAAAGTGTCAGTATCA |
| *cox3-nad4* | 4-C3-J-1.15a/4-N4-N-1.15b | TTCGTCAGACGCAAAACTTAG/TTTTGGCTTTTAGTTCTTTAGGA |
| *nad4-nad1* | N4-J-8944/Nl-N-12595 | GGAGCTTCAACATGAGCTTT/GTAGCATTTTTAACTTTATTAGAACG |
| *rrnL-rrnS* | LR-J-12887/SR-N-14588 | CCGGTCTGAACTCAGATCACGT/AAACTAGGATTAGATACCCTATTAT |
| *nad1-rrnL* | 4-N1-J-1.15a/4-LR-N-1.15c | CTCTCTTCATAAGAAACAGATTGTG/ATGGGACGAGAAGACCCTATA |
| *rrnS* | 4-13940-J/SR-N14745 | ATTTACTATGTTACGACTTTCCTCAC/GTGCCAGCAGYYGCGGTTANAC |
| *rrnS-nad2* | 4-14178-F-6.1/4-400-N-6.1 | ACTCTCCAAACAGCAATACACA/AATGAATGATATTGTTGAGATTTC |

Note: F, forward; R, reverse.
