# Supplementary material for: Phylogenetic analysis of the mitochondrial genomes in bees (Hymenoptera: Apoidea: Anthophila)
Source: PLoS One. 2018 Aug 9;13(8):e0202187. doi: 10.1371/journal.pone.0202187 (PMC6084986; doi:10.1371/journal.pone.0202187)
Supplement: S2 Table — (DOCX) [file pone.0202187.s003.docx]

**Table S2. List of 64** **species in this study.**

| Family | Species | Total Size (bp) | Genbank no.1 | Genbank no.2 | Genbank no.3 | Genbank no.4 |
| --- | --- | --- | --- | --- | --- | --- |
| Melittidae | *Rediviva intermixta* | 16875 | KR864834 |  |  |  |
| Andrenidae | *Andrena angustior* | 15252 | KT164658 |  |  |  |
|  | *Andrena bicolor* | 15422 | KT164666 |  |  |  |
|  | *Andrena camellia* | 15065 | KX241615 |  |  |  |
|  | *Andrena cineraria* | 17069 | KT164628 |  |  |  |
|  | *Andrena dorsata* | 16333 | KT164633 |  |  |  |
|  | *Andrena flavipes* | 15074 | KT164679 |  |  |  |
|  | *Andrena fulva* | 15318 | KT164623 |  |  |  |
|  | *Andrena labiata* | 15074 | KT164613 |  |  |  |
|  | *Andrena minutula* | 15302 | KT164675 |  |  |  |
|  | *Andrena nigroaenea* | 15376 | KT164665 |  |  |  |
|  | *Andrena nitida* | 14996 | KT164636 |  |  |  |
|  | *Andrena semilaevis* | 16459 | KT164629 |  |  |  |
|  | *Andrena subopaca* | 14747 | KT164612 |  |  |  |
|  | *Andrena chrysosceles* | 15692 | KT164687 | KT164688 | KT164602 |  |
|  | *Andrena haemorrhoa* | 16139 | KT164645 | KT164635 |  |  |
| Halictidae | *Halictus rubicundus* | 13578 | KT164668 | KT164656 |  |  |
|  | *Seladonia tumulorum* | 15268 | KT164609 |  |  |  |
|  | *Sphecodes ephippius* | 12890 | KT164611 | KT164659 |  |  |
|  | *Lasioglossum calceatum* | 12527 | KT164620 | KT164643 |  |  |
|  | *Lasioglossum fulvicorne* | 13537 | KT164615 | KT164650 | KT164652 | KT164673 |
|  | *Lasioglossum laevigatum* | 10564 | KT164625 | KT164642 | KT16466 |  |
|  | *Lasioglossum lativentre* | 13069 | KT164682 |  |  |  |
|  | *Lasioglossum leucopus* | 11172 | KT164610 | KT164662 |  |  |
|  | *Lasioglossum malachurum* | 12868 | KT164632 | KT164674 | KT164678 |  |
|  | *Lasioglossum minutissimum* | 13007 | KT164626 | KT164637 | KT164664 |  |
|  | *Lasioglossum morio* | 12962 | KT164604 | KT164605 | KT164608 |  |
|  | *Lasioglossum parvulum* | 11588 | KT164648 | KT164671 | KT164680 |  |
|  | *Lasioglossum pauxillum* | 12973 | KT164624 | KT164647 |  |  |
|  | *Lasioglossum punctatissimum* | 15408 | KT164603 | KT164606 | KT164607 |  |
|  | *Lasioglossum villosulum* | 11238 | KT164651 | KT164654 | KT164667 |  |
|  | *Lasioglossum xanthopus* | 11320 | KT164683 |  |  |  |
| Colletidae | *Colletes gigas* | 15885 | KM978210 |  |  |  |
|  | *Hylaeus dilatatus* | 15475 | NC_026468 |  |  |  |
|  | *Hylaeus confusus* | 13478 | KT164621 |  |  |  |
| Apidae | *Apis andreniformis* | 17529 | KF736157 |  |  |  |
|  | *Apis cerana* | 15712 | KM244704 |  |  |  |
|  | *Apis florea* | 17694 | JX982136 |  |  |  |
|  | *Apis mellifera intermissa* | 16336 | KM458618 |  |  |  |
|  | *Apis mellifera ligustica* | 16343 | NC_001566 |  |  |  |
|  | *Apis mellifera scutellata* | 16411 | KJ601784 |  |  |  |
|  | *Apis mellifera syriaca* | 15428 | KP163643 |  |  |  |
|  | *Apis mellifera* | 15348 | KT164631 | KT164619 |  |  |
|  | *Bombus hypocrita sapporensis* | 15769 | AP017370 |  |  |  |
|  | *Bombus ignitus* | 16434 | DQ870926 |  |  |  |
|  | *Bombus lapidarius* | 17817 | KT164641 |  |  |  |
|  | *Bombus hortorum* | 15487 | KT164638 | KT164676 | KT164614 | KT164627 |
|  | *Bombus lucorum* | 13639 | KT164640 | KT16468 | KT164657 |  |
|  | *Bombus pascuorum* | 13973 | KT164630 |  |  |  |
|  | *Bombus pratorum* | 14384 | KT164686 | KT164685 | KT164684 |  |
|  | *Bombus sylvestris* | 14726 | KT164655 | KT164644 | KT164622 |  |
|  | *Bombus terrestris* | 14386 | KT164677 | KT164618 | KT164616 |  |
|  | *Melipona bicolor* | 14422 | AF466146 |  |  |  |
|  | *Melipona scutellaris* | 14862 | KP202303 |  |  |  |
|  | *Nomada flavoguttata* | 14854 | KT164617 |  |  |  |
|  | *Nomada goodeniana* | 15201 | KT164660 |  |  |  |
|  | *Nomada fabriciana* | 14608 | KT164663 | KT164672 |  |  |
|  | *Nomada flava* | 15055 | KT164670 |  |  |  |
|  | *Nomada ruficornis* | 12361 | KT164639 | KT164649 |  |  |
| Megachilidae | *Osmia bicornis* | 13507 | KT164634 | KT164653 | KT164669 |  |
|  | *Megachile sculpturalis* | 16581 | KT223644 |  |  |  |
|  | *Megachile strupigera* | 15193 | KT346366 |  |  |  |
| Crabronidae | *Philanthus triangulum* | 16029 | JN871914 |  |  |  |
| Vespidae | *Abispa ephippium* | 16953 | EU302588 |  |  |  |
