# Supplementary material for: Phylogenetic analysis of the mitochondrial genomes in bees (Hymenoptera: Apoidea: Anthophila)
Source: PLoS One. 2018 Aug 9;13(8):e0202187. doi: 10.1371/journal.pone.0202187 (PMC6084986; doi:10.1371/journal.pone.0202187)
Supplement: S3 Table — (DOCX) [file pone.0202187.s004.docx]

**Table S3. Nucleotide composition of the mitogenomes of 64 species.**

| Family | Species | complete/partial | A% | C% | G% | T% | A+T% | N% | AT skew | GC skew |
| --- | --- | --- | --- | --- | --- | --- | --- | --- | --- | --- |
| Melittidae | *Rediviva intermixta* | complete | 38.54 | 10.61 | 9.51 | 41.33 | 79.87 | 0.2 | -0.035 | -0.055 |
| Andrenidae | *Andrena angustior* | partial | 44.43 | 12.93 | 6.74 | 35.62 | 80.05 | 0.29 | 0.110 | -0.315 |
|  | *Andrena bicolor* | partial | 43.37 | 13.34 | 7.22 | 35.63 | 79 | 0.45 | 0.098 | -0.298 |
|  | *Andrena camellia* | complete | 45.95 | 14.7 | 6.72 | 32.63 | 78.58 | - | 0.170 | -0.373 |
|  | *Andrena cineraria* | partial | 43.8 | 13.68 | 7.04 | 35.02 | 78.82 | 0.46 | 0.111 | -0.320 |
|  | *Andrena dorsata* | partial | 41.98 | 16.21 | 8.08 | 33.33 | 75.31 | 0.4 | 0.115 | -0.335 |
|  | *Andrena flavipes* | partial | 42.94 | 17.99 | 7.91 | 31.15 | 74.09 | - | 0.159 | -0.389 |
|  | *Andrena fulva* | partial | 42.89 | 15.85 | 7.69 | 33.1 | 75.99 | 0.46 | 0.129 | -0.347 |
|  | *Andrena labiata* | partial | 43.59 | 13.69 | 7.32 | 35.38 | 78.97 | 0.01 | 0.104 | -0.303 |
|  | *Andrena minutula* | partial | 42.82 | 16.47 | 7.63 | 33.08 | 75.9 | - | 0.128 | -0.367 |
|  | *Andrena nigroaenea* | partial | 43.98 | 15.59 | 7.59 | 33.84 | 76.82 | 0.01 | 0.130 | -0.345 |
|  | *Andrena nitida* | partial | 43.77 | 14.96 | 7.13 | 33.85 | 77.62 | 0.3 | 0.128 | -0.354 |
|  | *Andrena semilaevis* | partial | 43.28 | 15.77 | 7.08 | 33.35 | 76.63 | 0.52 | 0.130 | -0.380 |
|  | *Andrena subopaca* | partial | 43.6 | 15.07 | 7.2 | 33.5 | 77.1 | - | 0.131 | -0.353 |
|  | *Andrena chrysosceles* | partial | 44.98 | 15.79 | 7.53 | 33.46 | 76.44 | 0.24 | 0.147 | -0.354 |
|  | *Andrena haemorrhoa* | partial | 44.6 | 13.45 | 6.72 | 35.21 | 79.81 | 0.02 | 0.118 | -0.334 |
| Halictidae | *Halictus rubicundus* | partial | 39.7 | 11.71 | 6.42 | 42.17 | 81.87 | - | -0.030 | -0.292 |
|  | *Seladonia tumulorum* | partial | 40.35 | 6.14 | 11.99 | 39.6 | 79.95 | 1.93 | 0.009 | 0.323 |
|  | *Sphecodes ephippius* | partial | 41.33 | 10.73 | 6.31 | 41.64 | 82.97 | - | -0.004 | -0.259 |
|  | *Lasioglossum calceatum* | partial | 41.87 | 11.09 | 6.03 | 40.42 | 82.29 | 0.6 | 0.018 | -0.296 |
|  | *Lasioglossum fulvicorne* | partial | 40.81 | 11.33 | 6.12 | 41.74 | 82.55 | - | -0.011 | -0.299 |
|  | *Lasioglossum laevigatum* | partial | 39.19 | 12.57 | 8.63 | 3808 | 77.99 | 0.8 | -0.980 | -0.186 |
|  | *Lasioglossum lativentre* | partial | 40.22 | 12.83 | 6.63 | 40.32 | 80.54 | - | -0.001 | -0.319 |
|  | *Lasioglossum leucopus* | partial | 40.78 | 14.04 | 7.04 | 38.1 | 78.88 | 0.05 | 0.034 | -0.332 |
|  | *Lasioglossum malachurum* | partial | 41.02 | 11.77 | 6.11 | 40.97 | 81.99 | 0.14 | 0.001 | -0.317 |
|  | *Lasioglossum minutissimum* | partial | 41.15 | 11.4 | 6.35 | 41.09 | 82.24 | - | 0.001 | -0.285 |
|  | *Lasioglossum morio* | partial | 41.04 | 13.45 | 6.36 | 38.96 | 80 | 0.18 | 0.026 | -0.358 |
|  | *Lasioglossum parvulum* | partial | 40.95 | 11.21 | 6.76 | 41.09 | 82.04 | - | -0.002 | -0.248 |
|  | *Lasioglossum pauxillum* | partial | 40.58 | 11.81 | 6.17 | 41.44 | 82.02 | - | -0.010 | -0.314 |
|  | *Lasioglossum punctatissimum* | partial | 39.1 | 13.4 | 8.46 | 38.32 | 77.42 | 0.71 | 0.010 | -0.226 |
|  | *Lasioglossum villosulum* | partial | 40.91 | 10.91 | 6.53 | 41.16 | 82.07 | 0.51 | -0.003 | -0.251 |
|  | *Lasioglossum xanthopus* | partial | 40.34 | 12.85 | 6.72 | 40.09 | 80.43 | - | 0.003 | -0.313 |
| Colletidae | *Colletes gigas* | complete | 42.06 | 7.43 | 6.38 | 44.13 | 86.19 | - | -0.024 | -0.076 |
|  | *Hylaeus dilatatus* | complete | 44.26 | 8.8 | 5.4 | 41.53 | 85.79 | 0.02 | 0.032 | -0.239 |
|  | *Hylaeus confusus* | partial | 44.2 | 10.88 | 6.52 | 37.79 | 81.99 | 0.61 | 0.078 | -0.251 |
| Apidae | *Apis andreniformis* | partial | 41.19 | 7.43 | 6.49 | 43.74 | 84.93 | 1.14 | -0.030 | -0.068 |
|  | *Apis cerana* | partial | 42.23 | 9.83 | 6.33 | 41.61 | 83.84 | - | 0.007 | -0.217 |
|  | *Apis florea* | complete | 44.7 | 8.82 | 5.07 | 41.42 | 86.12 | - | 0.038 | -0.270 |
|  | *Apis mellifera intermissa* | complete | 43.17 | 9.76 | 5.65 | 41.42 | 84.59 | - | 0.021 | -0.267 |
|  | *Apis mellifera ligustica* | complete | 43.21 | 9.61 | 5.54 | 41.65 | 84.86 | - | 0.018 | -0.269 |
|  | *Apis mellifera scutellata* | complete | 43.25 | 9.69 | 5.61 | 41.45 | 84.7 | - | 0.021 | -0.267 |
|  | *Apis mellifera syriaca* | complete | 42.88 | 9.97 | 5.85 | 41.3 | 84.18 | 0.01 | 0.019 | -0.260 |
|  | *Apis mellifera* | partial | 43.52 | 9.54 | 5.58 | 41.35 | 84.87 | - | 0.026 | -0.262 |
|  | *Bombus hypocrita sapporensis* | complete | 42.99 | 9.65 | 4.83 | 42.52 | 85.51 | 0.01 | 0.005 | -0.333 |
|  | *Bombus ignitus* | complete | 43.51 | 8.41 | 4.81 | 43.26 | 86.77 | - | 0.003 | -0.272 |
|  | *Bombus lapidarius* | partial | 44.03 | 9.67 | 4.44 | 41.7 | 85.73 | 0.16 | 0.027 | -0.371 |
|  | *Bombus hortorum* | partial | 43.17 | 9.4 | 5 | 42.2 | 85.37 | 0.23 | 0.011 | -0.306 |
|  | *Bombus lucorum* | partial | 42.58 | 9.44 | 5.36 | 42.17 | 84.75 | 0.45 | 0.005 | -0.276 |
|  | *Bombus pascuorum* | partial | 42.36 | 7.34 | 5.63 | 43.83 | 86.19 | 0.84 | -0.017 | -0.132 |
|  | *Bombus pratorum* | partial | 43.33 | 8.5 | 5.14 | 43.02 | 86.35 | 0.01 | 0.004 | -0.246 |
|  | *Bombus sylvestris* | partial | 42.89 | 9.35 | 4.6 | 42.28 | 85.17 | 0.88 | 0.007 | -0.341 |
|  | *Bombus terrestris* | partial | 42.74 | 9.79 | 5.19 | 41.99 | 84.73 | 0.29 | 0.009 | -0.307 |
|  | *Melipona bicolor* | partial | 44.04 | 8.31 | 4.97 | 42.68 | 86.72 | - | 0.016 | -0.252 |
|  | *Melipona scutellaris* | complete | 43.92 | 8.24 | 4.95 | 42.89 | 86.81 | - | 0.012 | -0.249 |
|  | *Nomada flavoguttata* | partial | 41.58 | 7.12 | 6.17 | 44.98 | 86.56 | 0.15 | -0.039 | -0.071 |
|  | *Nomada goodeniana* | partial | 40.75 | 6.85 | 6.91 | 44.93 | 85.68 | 0.55 | -0.049 | 0.004 |
|  | *Nomada fabriciana* | partial | 42.07 | 7.74 | 5.91 | 43.44 | 85.51 | 0.81 | -0.016 | -0.134 |
|  | *Nomada flava* | partial | 41.75 | 7.13 | 6.07 | 44.04 | 85.79 | 1.02 | -0.027 | -0.080 |
|  | *Nomada ruficornis* | partial | 41.75 | 7.26 | 6.7 | 44.01 | 85.76 | 0.52 | -0.026 | -0.040 |
| Megachilidae | *Osmia bicornis* | partial | 40.42 | 9.28 | 6.37 | 43.92 | 84.34 | 0.01 | -0.041 | -0.186 |
|  | *Megachile sculpturalis* | complete | 42.77 | 10.71 | 5.93 | 40.59 | 83.36 | 0.01 | 0.026 | -0.287 |
|  | *Megachile strupigera* | partial | 41.99 | 10.28 | 6.28 | 41.45 | 83.44 | - | 0.006 | -0.242 |
| Crabronidae | *Philanthus triangulum* | complete | 44.43 | 10.22 | 6.14 | 39.21 | 83.64 | - | 0.062 | -0.249 |
| Vespidae | *Abispa ephippium* | complete | 39.55 | 13.38 | 6.02 | 41.05 | 80.6 | - | -0.019 | -0.379 |

Note: N, means the nucleotides that were not sequenced accurately.
